# Prevalence, Risk Factors, and Antimicrobial Resistance Profiles of Thermophilic *Campylobacter* Species in Humans and Animals in Sub-Saharan Africa: A Systematic Review

**DOI:** 10.1155/2020/2092478

**Published:** 2020-01-14

**Authors:** Noel Gahamanyi, Leonard E. G. Mboera, Mecky I. Matee, Dieudonné Mutangana, Erick V. G. Komba

**Affiliations:** ^1^SACIDS Foundation for One Health, College of Veterinary Medicine and Biomedical Sciences, Sokoine University of Agriculture, P.O. Box 3015, Chuo Kikuu, Morogoro, Tanzania; ^2^School of Medicine, Muhimbili University of Health and Allied Sciences, P.O. Box 65001, Dar es Salaam, Tanzania; ^3^College of Science and Technology, University of Rwanda, P.O. Box 3900, Kigali, Rwanda

## Abstract

Thermophilic *Campylobacter* species are clinically important aetiologies of gastroenteritis in humans throughout the world. The colonization of different animal reservoirs by *Campylobacter* poses an important risk for humans through shedding of the pathogen in livestock waste and contamination of water sources, environment, and food. A review of published articles was conducted to obtain information on the prevalence and antimicrobial resistance (AMR) profiles of thermophilic *Campylobacter* species in humans and animals in sub-Saharan Africa (SSA). Electronic databases, namely, PubMed, Google Scholar, Research4life-HINARI Health, and Researchgate.net, were searched using the following search terms “thermophilic *Campylobacter*,” “*Campylobacter jejuni*,” “*Campylobacter coli*,” “diarrhea/diarrhoea,” “antimicrobial resistance,” “antibiotic resistance,” “humans,” “animals,” “Sub-Saharan Africa,” and “a specific country name.” Initially, a total of 614 articles were identified, and the lists of references were screened in which 22 more articles were identified. After screening, 33 articles on humans and 34 on animals and animal products were included in this review. In humans, Nigeria reported the highest prevalence (62.7%), followed by Malawi (21%) and South Africa (20.3%). For *Campylobacter* infections in under-five children, Kenya reported 16.4%, followed by Rwanda (15.5%) and Ethiopia (14.5%). The country-level mean prevalence in all ages and under-five children was 18.6% and 9.4%, respectively. The prevalence ranged from 1.7%–62.7% in humans and 1.2%–80% in animals. The most reported species were *C. jejuni* and *C. coli*. The AMR to commonly used antimicrobials ranged from 0–100% in both humans and animals. Poultry consumption and drinking surface water were the main risk factors for campylobacteriosis. The present review provides evidence of thermophilic *Campylobacter* occurrence in humans and animals and high levels of AMR in SSA, emphasizing the need for strengthening both national and regional multisectoral antimicrobial resistance standard surveillance protocols to curb both the campylobacteriosis burden and increase of antimicrobial resistance in the region.

## 1. Introduction

Diarrhoea remains the main cause of morbidity and mortality in low- and middle-income countries (LMICs) [1–3]. Worldwide, under-five children experience approximately 1.4 billion episodes of diarrhoea each year, with several medical checks, hospitalizations, and around two million deaths. Over 78% of diarrhoea cases are found in the LMICs [4]. The burden of diarrhoeal diseases is complicated by the lack of appropriate case management [5], limited ability to detect the aetiologies [6], and antimicrobial resistance [7].

The most common aetiologies of diarrhoea include bacteria such as *Escherichia coli, Vibrio cholerae*, *Campylobacter jejuni*, *Salmonella* spp., *Aeromonas* spp., and *Yersinia enterocolitica*; viruses mainly rotavirus, norovirus, sapovirus, and adenovirus; and protozoa largely *Entamoeba histolytica*, *Giardia* spp., and *Cryptosporidium* spp. [8, 9]. Of the bacterial aetiologies, *Campylobacter* is a leading cause of gastroenteritis in both high-, middle-, and low- income countries, responsible for 400–500 million cases of diarrhoea each year [10]. The clinically important *Campylobacter* species are *C. jejuni* and *C. coli*, which are responsible for about 98% of all human *Campylobacter* gastroenteritis cases [11, 12].

In most cases, campylobacteriosis does not require any antimicrobial therapy except in severe cases, especially in immune-deficient or immune-suppressed individuals [13, 14]. The recommended drugs are macrolides (mostly erythromycin), fluoroquinolones (mainly ciprofloxacin), and tetracycline [10, 15, 16]. Nevertheless, there is an escalating number of *Campylobacter* isolates resistant to these drugs [17, 18] due to the immeasurable and misuse of antimicrobials [19], not only in animals but also in humans [20]. Several factors have been associated with occurrence of *Campylobacter* infections. They include consumption of different food items like undercooked poultry meat and pork, red meat at barbecue, grapes, and drinking unpasteurized milk, having a chronic illness [21–23], drinking contaminated water, type of water source, animal contact, young age, eating prepared salad, latrine usage, bottle feeding, and nutritional status [24–26]. There is a wide range of natural reservoirs for *Campylobacter* including chicken and other poultry, wild birds, pigs, dogs, cats, sheep, and cows [27, 28]. Consequently, colonization of different reservoirs by *Campylobacter* poses an important risk for humans through shedding of the pathogen in livestock waste and water sources contamination, environment, and food [29, 30].

In LMICs, studies on thermophilic *Campylobacter* species are few due to limited capacity in laboratory diagnosis [31] and lack of surveillance of enteric diseases [32]. The objective of this review was to gather information on the prevalence, risk factors, and antimicrobial resistance profiles of thermophilic *Campylobacter* species in humans and animals in SSA. The findings of this review are expected to provide evidence for policy formulation, prevention, and control of *Campylobacter* infections and increase awareness of the AMR issue.

## 2. Methods

**Figure 1:**
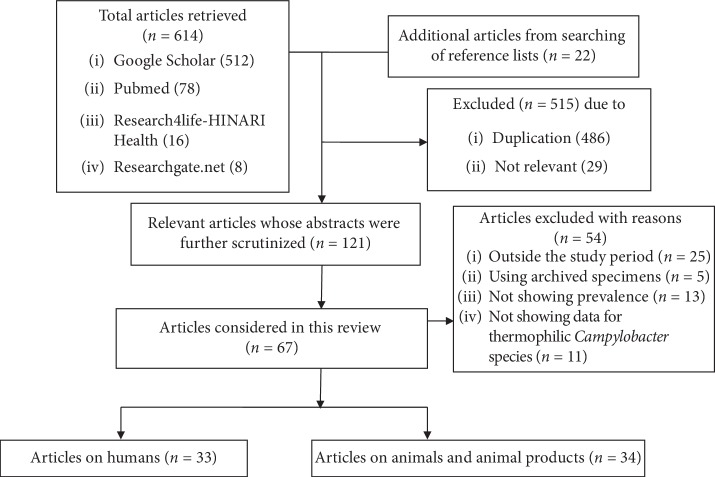
Flowchart showing article selection process.

The data were collected by searching articles published in English from electronic databases, namely, PubMed, Google Scholar, Research4life-HINARI Health, and Researchgate.net. The search terms were “thermophilic *Campylobacter*,” “*Campylobacter jejuni*,” “*Campylobacter coli*,” “diarrhea/diarrhoea,” “antimicrobial resistance,” “antibiotic resistance,” “humans,” “animals,” “Sub-Saharan Africa,” and “a specific country name.” Initially, a total of 614 articles were identified, and the lists of references were screened in which 22 more articles were identified. After screening, 33 articles on humans and 34 on animals and animal products were included in this review (Figure 1). The reviewed articles were those published from 1997 to 2018. During the review process, the data extracted included title, country, sex and age distribution, sample size, isolation and identification methods, isolation rates, and antimicrobial resistance profiles. Articles for which the sample size was not shown or which used archived *Campylobacter* cultures were excluded from this review.

## 3. Results

### 3.1. *Campylobacter* Infections in Humans

Of the 47 SSA countries [33], data on human campylobacteriosis were available from 15 (31.9%) countries. The prevalence of thermophilic *Campylobacter* in humans was reported in 33 articles (Table 1). Nigeria reported the highest overall prevalence of thermophilic *Campylobacter* (62.7%); followed by Malawi (21%) and South Africa (20.3%). Kenya reported the highest prevalence (16.4%) of *Campylobacter* infections in under-five children; followed by Rwanda (15.5%) and Ethiopia (14.5%). The mean prevalence in all ages and under-five children was 18.6% and 9.4%, respectively. Burkina Faso and Mozambique had the lowest prevalence of campylobacteriosis for all ages (2.3%) and under-five (1.7%), respectively. Of the 33 articles reviewed, 16 (48.5%) presented data on distribution of *Campylobacter* infections by sex but the difference was not statistically significant. Of these 16 articles, campylobacteriosis was more prevalent among males (22.7%; *n* = 3966) than females (17.7%; *n* = 3705). Culture methods on selective media, biochemical tests, molecular, and biotyping techniques were used for identification of *Campylobacter* (Table 1). Of the 33 articles, 27 studies were carried out at clinical settings (hospitals and health centres) while 6 were community-based studies. Probability sampling methods were adopted in 5 articles while the remaining used convenience sampling. Although *C. jejuni* and *C. coli* were isolated in the mentioned articles, 15 articles reported other enteric pathogens as probable aetiologies of diarrhoea. Furthermore, more than 85% of the articles considered diarrhoeic cases while the remaining included even asymptomatic participants.

Of the 33 articles, only four reported on risk factors of campylobacteriosis in humans. In Tanzania, *Campylobacter* infections were associated with sex, young age, poultry meat consumption, and eating of salads [26, 38]. In Ethiopia, human campylobacteriosis was significantly associated with nonuse of latrines, water source, drinking unboiled water, bottle feeding, nutritional status, and exposure to domestic animals including cats, dogs, poultry, and pigeons [25]. In Burkina Faso, *Campylobacter* infections were most common among under-fives and those aged 21–40 years with more pet contacts [57].

### 3.2. *Campylobacter* spp. in Animals and Contamination of Animal Products

Of the 34 articles from which data on animals were extracted, 17 collected faeces from live animals, while 16 collected samples from meat or caeca at abattoirs. In 2 articles, samples were collected from both markets and abattoirs. Probability sampling methods were used in 6 articles while the remaining used convenience sampling.

Data on *Campylobacter* in cattle were obtained from ten articles published from studies conducted in six countries. The overall mean prevalence was 17.6% and *C. jejuni* had higher prevalence (70%) than *C. coli* (23.5%). The highest [64] and the lowest overall prevalence [52] were reported from Tanzania. Furthermore, Tanzania and Ghana showed higher prevalence for *C. jejuni* and *C. coli*, respectively (Table 2).

Data on *Campylobacter* in goats were reported in three articles from three different countries. The overall mean prevalence was 31.2%, and *C. jejuni* presented with a higher prevalence (56.2%) than *C. coli* (38.5%). The highest and lowest prevalence were reported from the Democratic Republic of Congo (DRC) [83] and Ghana [83], respectively. Ethiopia [70] and DRC [83] had the highest frequencies for *C. jejuni* and *C. coli*, respectively (Table 2). For sheep, data were reported in four articles from three countries. The overall mean prevalence was 31.8%, with *C. jejuni* being reported at a higher frequency (56.7%) than *C. coli* (35.4%). The highest and lowest prevalence were reported from Ethiopia [70] and Ghana [70], respectively. Ethiopia [70] and Tanzania [75] had the highest prevalence for C. *jejuni* and C. *coli*, respectively (Table 2).

Data on presence of thermophilic *Campylobacter* in pigs were available from six articles from five countries. The overall mean prevalence was 45.5% and contrary to other animals, *C. coli* occurred at a higher prevalence (70.1%) than *C. jejuni* (27.2%). The highest and lowest prevalence were reported from Nigeria [60] and South Africa [78], respectively. Ethiopia [69] had both higher and lower values for *C. jejuni* and *C. coli* (Table 2).

Data on thermophilic *Campylobacter* in chickens were obtained from 11 articles from five different countries. In this review, the number of articles on chickens was the highest compared to other reservoirs. The overall mean prevalence was 62.6% which was the highest in all animal reservoirs documented in this review. *Campylobacter jejuni* was reported in higher prevalence (81.0%) than *C. coli* (18.1%). The highest and lowest prevalence rates were reported in Ethiopia [70] and South Africa [18], respectively (Table 2).

As regards to animal products, data on cattle meat were reported in three articles from three countries. The overall mean prevalence was 5.5%, and *C. jejuni* had higher prevalence (95.2%) than *C. coli* (4.8%). The highest and lowest prevalence rates were reported in Ethiopia [72] and Kenya [73], respectively. For cattle carcasses, data were reported by two articles from two countries with a mean prevalence of 15.9%. Ghana [68] reported a higher prevalence of *C. jejuni* while Tanzania [74] observed a higher prevalence of *C. coli* (Table 2).

Data on sheep meat were reported by a single article from Ethiopia [72] with the prevalence of 10.5%. In sheep carcasses, the mean prevalence was 23.3% computed using two articles from two countries. Ghana [68] showed a higher prevalence of *C. jejuni* while Ethiopia [76] reported a higher prevalence of *C. coli*. In pork, the prevalence was 8.5% in one article from Ethiopia [72] with *C. coli* being more prevalent than *C. jejuni*. In pig carcasses, the prevalence was 36.3% from one article reporting a study carried out in Ghana [68]. In chicken meat, the mean prevalence was 49.4% reported by two articles from two countries. Dadi and Asrat in a study conducted in Ethiopia [72] indicated a higher prevalence for *C. jejuni* while a study in Kenya [73] found a higher prevalence for *C. coli*. For chicken carcasses, the prevalence was 50% from one article in Burkina Faso [79] and all isolates were *C. jejuni*. In goat meat, the mean prevalence was 22.5% reported by only one article from Ethiopia [72]. In goat carcasses, the mean prevalence was 16.7% reported by two articles from two countries. A study conducted in Ghana [76] reported a higher prevalence for *C. jejuni* while that in Ethiopia [76] found a higher prevalence for *C. coli* (Table 2).

The overall prevalence of thermophilic *Campylobacter* in cats [84] and dogs [84, 85] were 18.3% and 20%, respectively. Of the reviewed articles, some presented data on companion, wild, and other animals (Table 3).

### 3.3. Antimicrobial Resistance Profiles of *C. jejuni* and *C. coli* in Humans and Animals

**Figure 2:**
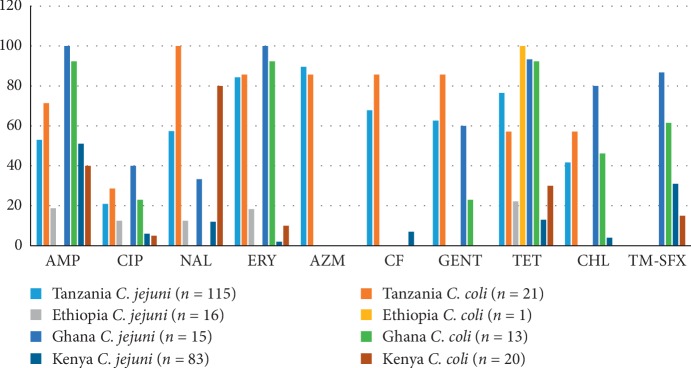
Antimicrobial resistance data in humans by the disk diffusion method.

In humans, the AMR profiles, determined using disk diffusion, were available in 4 articles from four different countries (Figure 2), while the remaining did not specify the species. The antimicrobials considered in this review for the ease of comparison were ampicillin (AMP), erythromycin (ERY), tetracycline (TET), cefalotin (CF), nalidixic acid (NAL), azithromycin (AZM), gentamicin (GEN), ciprofloxacin (CIP), chloramphenicol (CHL), and trimethoprim-sulfamethoxazole (TM-SFX).

The percentage of antimicrobial resistant isolates ranged from 2–100% for *C. jejuni* and 0–100% for *C. coli*. The AMR data for CIP and ERY, which are drugs of choice for treating *Campylobacter* infections, showed that Ghana [61] and Tanzania [26] reported higher values for both *C. jejuni* and *C. coli*. Resistance of *Campylobacter jejuni* to GEN was similar for both Tanzania and Ghana while for *C. coli*, it was higher in Tanzania compared eith that of Ghana [26, 61]. Higher frequencies of resistance were also reported for TET and AMP which have been in use for many years. In general, higher levels of AMR were reported in *C. jejuni* than *C. coli*.

**Figure 3:**
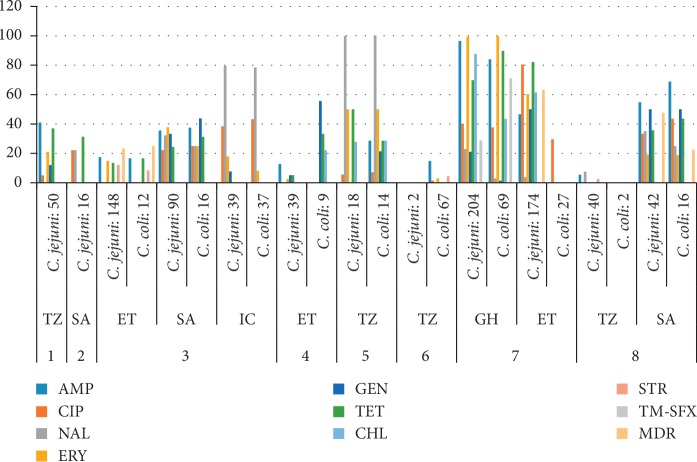
Antimicrobial resistance data in animals by the disk diffusion method. 1: duck, 2: sea birds, 3: chicken, 4: raw meat, 5: laboratory and farm animals, 6: pig, 7: food animals, 8: cattle; TZ: Tanzania, SA: South Africa, ET: Ethiopia, IC: Ivory Coast, GH: Ghana.

In animal and animal products, the following antimicrobials were used in the reviewed articles: chloramphenicol (CHL), ampicillin (AMP), erythromycin (ERY), ciprofloxacin (CIP), nalidixic acid (NAL), streptomycin (STR), tetracycline (TET), gentamicin (GEN), and trimethoprim-sulfamethoxazole (TM-SFX) (Figure 3).

In animals, the percentage of resistant isolates varied from 0–100%. Resistance to CIP was in the range of 0–80.5% and 0–68.8% for *C. jejuni* and *C. coli*, respectively. Resistance to ERY varied from 0–99.5% and 0–100% for *C. jejuni* and *C. coli*, respectively. Resistance to GEN was <55.6% for both *C. jejuni* and *C. coli*. The highest resistance to most of the drugs was seen in Ghana [68] while the lowest resistance was observed in Tanzania [74, 89]. Resistance to nalidixic acid was high for both *C. jejuni* and *C. coli* in a study conducted in Tanzania [75]. Data on multidrug resistance were available from three studies in which values ranged from 23.3% to 63.3% for *C. jejuni* [18, 59, 74] and from 0–25% for *C. coli* [59, 70]. There were variations in resistance levels to commonly used antimicrobials in animal species depending on the species tested.

## 4. Discussion

The overall mean prevalence of thermophilic *Campylobacter* in humans ranged from 9.6–18.5% and is within the ranges reported elsewhere in LMICs [31] and in Poland [90]. However, the prevalence was higher than that reported from Korea [91], and was lower than that reported from the USA [92]. This variation may be attributed to the fact that campylobacteriosis is hyperendemic in LMICs probably due to poor sanitation and close proximity of humans and domestic animals [31]. The risk factors for human infections highlighted in this review partly explain this. They include consumption of poultry meat, drinking surface water, and animal contact, which is in agreement with other studies with consumption of poultry being the major risk factor [24, 93].

The prevalence of thermophilic *Campylobacter* in animals varied between 1.2% and 80%. The mean prevalence recorded in chickens (60.3%) concurs with findings from other LMICs such as Thailand [94], Sri Lanka [95], and Vietnam [96]. The mean prevalence of thermophilic *Campylobacter* in pigs was comparable to what was reported in Spain and Vietnam [30, 96] but lower than those reported in Norway and the Netherlands [97, 98]. The prevalence of *Campylobacter* in goats and sheep was slightly higher than the prevalence reported in Germany and Trinidad [99, 100] but lower than the prevalence reported in Spain [30]. The prevalence in cattle (17.6%) was lower than those reported in the USA and Iran [101, 102] but higher than the prevalence reported in another paper in the USA [103].

Although thermophilic *Campylobacter* species are frequently isolated from animal faeces, this review showed that they are also present in considerable amounts in a number of animal products. The reported prevalence of *Campylobacter* in cattle and goat carcasses in sub-Saharan Africa was higher compared to the prevalence in Poland for cattle [104] and in Canada for goat [105]. The contamination of carcasses may result from contact with gut contents during manual skin removal, cleaning, and processing in the slaughter house [106]. The prevalence rates in beef, pork, and mutton were slightly higher compared to those observed in other countries [107–109]. The variation could be influenced by the differences in husbandry practices which determine exposure of the animals to the bacteria. Partly, this could also be attributable to slaughter and animal product handling practices which enhance the contamination of the products.

*Campylobacter jejuni* and *C. coli* were the most frequently encountered species from both human and animals. Similar observations have been reported by other authors [30, 110]. The predominance of *C. jejuni* in various animals, other than pigs, in sub-Saharan Africa has been previously reported [31, 111]. The possible explanation is that most of the studies rely on culture and biochemical tests which may not correctly identify some species. Another reason is the use of selective media containing antibiotics to which some other *Campylobacter* species are sensitive to. Furthermore, higher incubation temperatures may limit the growth of some thermophilic *Campylobacter* species like *C. lari* and *C. upsaliensis* [112, 113].

In pigs, *C. coli* showed higher prevalence (67.4%) than *C. jejuni* (27.2%) which is in agreement with reports in Canada and the USA that *C. coli* is a normal flora of pigs' intestines [114, 115]. Furthermore, some studies show that *C. jejuni* and *C. coli* may cohabit in pigs but usually *C. jejuni* is always present in lower frequencies than *C. coli* [116, 117].

The results on AMR in both humans and animals highlight that resistance to mostly used antimicrobials is frequent. The resistance ranged from 0 to 100%, and higher resistance rates were reported in *C. jejuni* than in *C. coli*. The antimicrobials to which resistance was high included AMP, TET, ERY, and TET. The findings concur with the reports from other studies in both LMICs and high-income countries showing an increment in the number of *Campylobacter* strains resistant to most of the antimicrobials used in treating human campylobacteriosis [118–120]. The increase in resistance to most antimicrobial agents and emergence of MDR isolates could be associated with extensive use of antimicrobials not only as therapeutic agents for human infections [20] but also for prophylaxis and growth promotion in animal husbandry [68]. However, there are challenges in surveillance, differences in design and predominance of the disk diffusion method and not using globally accepted methods. These may cause differences within and between countries and certainly limit comparability with data reported in other parts of the world. The resistance to TET was comparable with the findings reported from Poland [121] and the USA [122] and the pooled estimate prevalence worldwide (94.3%) [120]. This resistance may be due to wide use of tetracycline in both human and veterinary medicine [20]. The proportion of isolates resistant to macrolides (ERY) ranged from 0 to 100% in both humans and animals for *C. jejuni* while the range was from 0 to 92.3% for *C. coli*. The frequency of isolates resistant to fluoroquinolone was relatively lower in humans which is comparable to rates described in Western Europe [118, 121]. The resistance to both erythromycin and ciprofloxacin is of public health concern as there are currently limited options in the choice of treatment of *Campylobacter* infections. The proportion of multidrug resistance (MDR) isolates varied between 23.3 and 63.3% (Figure 3) which falls within the range of 37–90% from studies in China, Korea, and France [123–125].

There are no internationally agreed criteria of susceptibility testing and breakpoint assessment for *Campylobacter* spp. [126]. Therefore, it is difficult to interpret the available data and draw conclusion. Several laboratory standards have been applied for the susceptibility testing of *Campylobacter* species. Although disk diffusion was used in some studies, it should be used only as a screening method for resistance to erythromycin and ciprofloxacin [127].

## 5. Conclusion

This review indicates that *C. jejuni* and *C. coli* are frequently isolated from humans, food animals, and animal products in sub-Saharan Africa. Isolates from the different sources display varying degrees of resistance to commonly used antimicrobial agents. The findings of this review suggest that the disease burden due to thermophilic *Campylobacter* species in SSA is of public and economic importance. Therefore, routine diagnosis of *C. jejuni* and *C. coli*, appropriate use of antimicrobials, educating communities on hygienic practices, establishment of both national and regional multisectoral antimicrobial resistance standard surveillance protocols are necessary to curb both the campylobacteriosis burden, and increase of antimicrobial resistance in the region.

## Figures and Tables

**Table 1:** Prevalence of thermophilic *Campylobacter* spp. in humans in sub-Saharan Africa, 1997–2018.

Country	Age group (sample size)	Number of articles	Prevalence (%)	Detection method	References
Uganda	Children <5 (226)	1	9.3 (*C. jejuni*: 80.9%; *C. coli*: 4.8%)	Culture, biochemical	[34]
Tanzania	Children <5 (1,512)	5	8.8 (2.6–19) (*C. jejuni*: 89.2%; *C. coli*: 9.8%)	Culture, biochemical, Gram staining, molecular	[8, 35–38]
Kenya	Children <5 (2,550)	1	16.4	Culture, biochemical, serotyping	[39]
Rwanda	Children <5 (706)	1	15.5 (*C. jejuni*: 100%)	Molecular	[40]
Madagascar	Children <5 (5,620)	2	9.4 (9.3–9.5) (*C. jejuni*: 73.6; *C. coli*: 24.3%)	Culture, serotyping, molecular	[41, 42]
Burkina Faso	Children <5 (283)	1	2 (*C. jejuni*: 60%; *C. coli*: 40%)	Culture, molecular	[43]
Ethiopia	Children <5 (670)	2	14.5 (12.7–16.7) (*C. jejuni:* 71.1%; *C. coli*: 21.1%)	Culture, biochemical, Gram staining	[25, 44, 45]
Nigeria	Children <5 (1,311)	3	4.4 (0.5–8.2) (*C. jejuni*: 28%; *C. coli*: 72%)	Culture, biochemical, biotyping, Gram staining	[46–48]
Niger	Children <5 (260)	1	11.4 (*C. jejuni*: 100%)	Culture, biochemical, Gram staining	[49]
Mozambique	Children <5 (529)	1	1.7	Culture, biochemical, Gram staining	[50]
Cameroon	Children <5 (260)	1	9.6 (*C. jejuni*: 100%)	Culture, biochemical, Gram staining	[51]
Botswana	Under 15 years	1	14	Molecular	
Tanzania	All ages (2,487)	4	11.1 (1.9–21.6) (*C. jejuni*: 93.3%; *C. coli*: 6.1%)	Culture, biochemical, Gram staining, molecular	[26, 52–54]
Kenya	All ages (4,274)	2	9.2 (8.5–9.8) (*C. jejuni*: 76.2; *C. coli*: 12.7%)	Culture	[55, 56]
Burkina Faso	All ages (1,246)	1	2.3 (*C. jejuni*: 51.8%; *C. coli*: 13.8%)	Culture, biochemical, Gram staining	[57]
Ethiopia	All ages (640)	2	9.8 (8–11.6) (*C. jejuni*:94.1%; *C. coli*: 5.9%)	Culture, biochemical, Gram staining	[58, 59]
Nigeria	All ages (150)	1	62.7 (*C. jejuni*: 24.5%; *C. coli*: 62.3%)	Culture, biochemical, Gram staining	[60]
Ghana	All ages (202)	1	17.3 (*C. jejuni*: 42.8%; *C. coli*: 37%)	Culture, biochemical, Gram staining	[61]
Malawi	All ages (1,941)	1	21 (*C. jejuni*: 85%; *C. coli*: 14%)	Molecular	[62]
South Africa	All ages (565)	1	20.3 (*C. jejuni*: 85%; *C. coli*: 15%)	Culture, biochemical, molecular	[63]

**Table 2:** Prevalence of *Campylobacter* spp. in domestic animals and animal products.

Animal type	Sample type	Country	Overall prevalence	*C. jejuni* (%)	*C. coli* (%)	References
Cattle	Faeces	South Africa	19.3	72.4	27.6	[18]
		Nigeria	18.5	80	20	[65]
			12.9	65.1	23	[66]
		Tanzania	2.3	100	0	[52]
			5.6	83.3	16.7	[67]
			32.5	65.5	27.3	[64]
		Ghana	13.2	25	43.8	[68]
		Ethiopia	12.7	53.8	38.5	[69]
			48	75.3	17.6	[70]
		Mozambique	11	80	20	[71]
*Average*			**17.6**	**70**	**23.5**	

Cattle	Meat	Tanzania	2.8	100	0	[67]
		Ethiopia	6.2	85.7	14.3	[72]
		Kenya	2	100	0	[73]
*Average*			**5.5**	**95.2**	**4.8**	

Cattle	Carcasses	Tanzania	3.7	75	25	[67]
			9.5	62.5	29.2	[74]
		Ghana	34.5	84.2	13.1	[68]
*Average*			**15.9**	**73.9**	**22.4**	

Sheep	Faeces	Tanzania	31.6	55.6	44.4	[75]
		Ethiopia	38	59.3	40.7	[69]
			39	84.6	15.4	[70]
		Ghana	18.6	27.2	40.9	[68]
*Average*			**31.8**	**56.7**	**35.4**	

Sheep	Carcasses	Ethiopia	10.6	73.9	26.1	[76]
		Ghana	35.9	92.8	0	[68]
*Average*			**23.3**	**83.4**	**13.1**	

Sheep	Meat	Ethiopia	10.5	83.3	0	[72]
Pig	Faeces	Nigeria	92.7	14	78.7	[60]
		Ethiopia	50	0	100	[69]
		Tanzania	66.7	81.8	18.2	[77]
			32.5	2.7	91.9	[64]
		Ghana	28.7	48.2	48.2	[68]
		South Africa	2.3	16.7	83.3	[78]
*Average*			**45.5**	**27.2**	**70.1**	

Pig	Carcasses	Ghana	36.3	28.4	10.8	[68]
Pig	Pork	Ethiopia	8.5	25	50	[72]
Chicken	Faeces	Burkina Faso	68	70	30	[79]
		Tanzania	69.8	91.2	8.8	[53]
			42.5	87.1	12.9	[38]
			77.8	91.1	7.3	[54]
		South Africa	35.3	84.9	15.1	[18]
			49.7	100	0	[80]
			54.8	54.8	40.2	[81]
		Ethiopia	72.7	92.5	7.5	[59]
			68.1	80.8	16.2	[69]
			86.6	86.9	11.9	[70]
		Ivory Coast	63.8	51.3	48.7	[82]
*Average*			**62.6**	**81**	**18.1**	

Chicken	Colon	South Africa	14.2	68.8	31.2	[78]
Chicken	Carcasses	Burkina Faso	50	100	0	[79]
Chicken	Meat	Ethiopia	21.7	84	8	[72]
		Kenya	77	59	39	[73]
*Average*			**49.4**	**71.5**	**23.5**	

Goat	Faeces	DRC	41.7	32.7	59.4	[83]
		Ghana	18.5	36	56	[68]
		Ethiopia	33.3	100	0	[70]
*Average*			**31.2**	**56.2**	**38.5**	

Goat	Carcasses	Ethiopia	9.4	70.6	29.4	[76]
		Ghana	23.9	81.3	0	[68]
*Average*			**16.7**	**76**	**14.7**	

Goat	Meat	DRC	37.3	21.3	74.7	[83]
		Ethiopia	7.6	71.4	28.6	[72]
*Average*			**22.5**	**46.4**	**51.7**	

Cattle	Milk	Tanzania	13.4	55.3	31.6	[74]

**Table 3:** Prevalence of *Campylobacter* spp. in companion, wild, and other animals.

Animal type	Specimen	Country	Overall prevalence	*C. jejuni* (%)	*C. coli* (%)	References
*Companion animals*						
Cat	Faeces	Nigeria	18.3	21.1		[66]
Dog	Faeces	Nigeria	27.7	23.1	0	[66]
			12.3	53.8	30.8	[85]
		*Average*	**20**	**38.5**	**15.4**	

*Other animals*						
Crow	Faeces	Tanzania	72.8	93.8	6.2	[53]
Duck	Faeces	Tanzania	80	81.5		[86]
Greater crested tern	Faeces	South Africa	16	15	1	[87]
Kelp gull	Faeces	South Africa	12.4	11.6	0.8	[87]
Quail	Caeca	Nigeria	31.1	81	19	[88]
Horse	Faeces	Tanzania	60	66.7	33.3	[75]
Guinea pig	Faeces	Tanzania	26.7	50	50	[75]
Rat	Faeces	Tanzania	1.2	66.7	33.3	[75]
